# Rumen Microbiota Transplantation Alleviates Gossypol Diet-Induced Reproductive, Liver, and Intestinal Damage in Male Mice

**DOI:** 10.3390/ani14152206

**Published:** 2024-07-30

**Authors:** Chen Zhang, Wenguang Lu, Huiru Liu, Lingwei Shen, Mengfan Zhu, Tangtang Zhou, Ling Zhang, Dingfu Xiao, Lijuan Chen

**Affiliations:** 1 The Biological Feedstuff Labaratory, College of Animal Science and Technology, Anhui Agricultural University, Hefei 230036, China; 2 Animal Nutritional Genome and Germplasm Innovation Research Center, College of Animal Science and Technology, Hunan Agricultural University, Changsha 410128, China; 3 Laboratory of Gastrointestinal Microbiology, National Center for International Research on Animal Gut Nutrition, Nanjing Agricultural University, Nanjing 210095, China

**Keywords:** rumen microbes, gossypol, microbiota transplantation, liver, sperm, intestine

## Abstract

**Simple Summary:**

The rumen microbiota exerts multiple physiological effects. We attempted to assess whether rumen microbiota transplantation improved the symptoms of gossypol poisoning. Male mice transplanted with the ruminant rumen microbiota of Hu sheep showed relief from reproductive and liver damage induced by a gossypol diet. Supplementing the rumen microbiota increased animal feed intake and enhanced intestinal structural integrity.

**Abstract:**

Ruminants exhibit stronger tolerance to gossypol, an anti-nutritional factor, compared to monogastric animals. We transplanted Hu sheep rumen microbiota into male mice to investigate the role of rumen microbiota in animal gossypol tolerance. Thirty specific-pathogen-free (SPF) male C57BL/6 mice were randomly divided into three groups: normal diet (CK group), gossypol diet (FG group), and rumen microbiota transplantation (FMT group, gossypol diet). The pathological changes in the liver and small intestine of the mice, the organ coefficient, and sperm parameters were analyzed. Alanine aminotransferase (ALT) and aspartate aminotransferase (AST) levels in the blood and lactate dihydrogen-X (LDH-X) levels in the testicular tissue were also measured. The results showed that body weight, feed intake, sperm concentration, sperm motility, and LDH-X levels in the FMT group increased (*p* < 0.05) compared with the FG group, while the enzyme activities of ALT, AST, and AST/ALT decreased (*p* < 0.05). In the FMT group, the injury to liver cells was alleviated, the structure of the small intestine was intact, and the villus height and the ratio of villus height to crypt depth (V/C) were higher than those in the FG group (*p* < 0.05). And there were no differences in various organ coefficients and sperm deformity rates among the three groups (*p* > 0.05), but compared with the FG group, mice in the FMT group showed tendencies closer to those in the CK group. Rumen microbiota transplantation relieved the reproductive toxicity and liver damage induced by gossypol in male mice and improved the tolerance of recipient animals to gossypol. Additionally, rumen microbes improved the intestinal structural integrity of recipients.

## 1. Introduction

Cotton meal (CM), a potential source of protein feed, can partially replace soybean meal and alleviate the shortage of protein feed in China [1]. However, gossypol in CM, an anti-nutritional factor, limits its widespread use in animal husbandry [2,3]. Gossypol is absorbed in the gastrointestinal tract of humans and animals [4] and gets accumulated [5], with the highest concentration in the liver [6], followed by the kidney, intestine, testis, plasma, stomach, and muscles [7,8,9,10]. Long-term CM consumption can lead to chronic poisoning due to gossypol accumulation, which can damage the reproductive system, inhibit sperm production, affect the sperm survival rate, disrupt estrus cycles in female animals, cause uterine atrophy, and affect pregnancy [11,12,13]. Compared with monogastric animals, ruminants are more tolerant of gossypol; monogastric animals, such as poultry and pigs [14,15,16], often exhibit poisoning symptoms after ingesting gossypol, while ruminants generally do not exhibit them [17,18]. Some studies suggest that the rumen microbes of ruminants may increase their tolerance to gossypol [19,20].

Ruminants’ rumen houses a complex microbiota which can degrade complex polysaccharides and convert indigestible plant mass into various nutrients for the host [21]. Rumen microbiota also can degrade certain plant toxins, such as mimosine (a toxic amino acid found in plants of the genera *Leucaena* and *Mimosa*), 3,4-dihydroxy pyridine, sodium monofluoroacetate (a toxic compound in *Amorimia* spp.), and tannins, to reduce the damage caused by these toxins to the body [22]. And the rumen microbiome structure of adult ruminants is highly similar [23]; most bacteria in the rumen belong to Firmicutes, Bacteroidetes, and Proteobacteria [24,25]. Among these, Bacteroidetes and Firmicutes are the two dominant phyla in the sheep rumen, followed by Proteobacteria. The Prevotellaceae and Lachnospiraceae are the dominant families, and the *Ruminococcus* and *Prevotella* are the dominant genera [26]. Wang et al. indicate that gossypol addition affects the molar ratio of acetic, isobutyric, butyric, and isovaleric acids in sheep rumen fluid but the diversity of bacteria and relative abundance of dominant bacteria in rumen fluid remain unaffected [27]. The relationship between rumen microbes and gossypol tolerance in animals remains unclear [20]. It may be possible to study the role of rumen microbes in animal gossypol tolerance by transplanting rumen microbes into monogastric animals.

Fecal microbiota transplant (FMT) was originally a method for treating intestinal diseases. In recent years, microbiota transplantation technology represented by FMT has expanded from clinical medicine to animal husbandry. Using FMT to introduce the microbiota of healthy donor animals or specific functional microbiota into a recipient’s gut can rapidly and effectively improve the gut health of recipient animals and positively affect the immune function, metabolic function, and production performance [28,29]. Therefore, we used FMT to transplant the rumen microbiota into mice fed a gossypol diet. By analyzing the growth performance, organ coefficients, liver function, reproductive function, and small intestine structure of the mice, we aimed to study the role of the rumen microbiota in animal gossypol tolerance.

## 2. Material and Methods

### 2.1. Feeding of Mice and FMT

Fresh rumen fluid (collected from healthy Hu sheep, provided by the Experimental Farm and Pasture, Yangzhou University) was filtered using four layers of sterile gauze and centrifuged at 3420× *g* to collect the bacterial precipitate [30,31]. According to the description by Nanthini et al., the bacterial precipitate was pre-frozen at −80 °C for 48 h, then lyophilized at −45 °C, and stored at −80 °C [30]. The lyophilized precipitate was resuspended in an equal volume of 0.5% sterile carboxymethylcellulose sodium (CMC-Na) solution to obtain the oral FMT solution [31]. In the oral FMT solution, the viable rumen microbiota count was above 1 × 10^8^ cfu/mL (measured under conditions of 39 °C, anaerobic environment [31], and using beef extract peptone agar medium). Analysis by Shanghai Genesky Biotechnologies Inc. (Shanghai, China) showed that the dominant microbes in the rumen fluid at the phylum level included Bacteroidetes (39.13%), Firmicutes (32.31%), and Proteobacteria (19.01%). At the genus level, the dominant microbes included *Acinetobacter* (15.91%), RC9_gut_group (9.61%), *Prevotella* (7.20%), uncultured (5.19%), and *Ruminococcus* (3.11%).

Thirty adult male C57BL/6 mice (8 weeks old, body weight 34.08 ± 1.34 g, Zhejiang Hangzhou Ziyuan Experimental Animal Technology Co., Ltd., Hangzhou, China) were randomly divided into three groups (*n* = 10) and treated following the protocol shown in Table 1. The mice were raised under specific-free-pathogen (SPF) conditions (12:12 h light/dark cycle) with ad libitum access to food and water and fed adaptively for 7 days before experiments. Mice were subjected to gavage with 0.2 mL of CMC-Na or an oral FMT solution every other day for 10 weeks.

### 2.2. Clinical Information

Animals were checked daily for symptoms of poisoning, and their body weight and feed intake were assessed before each gavage. The average daily body weight and average daily feed intake (ADFI) of animals in each group were calculated.

### 2.3. Sample Collection

After the last measurement, the animals were made to fast for 8 h, and all mice were administered ether inhalation anesthesia. Whole blood was collected from the orbit and immediately placed on ice. Serum obtained by centrifugation at 8000× *g* at 4 °C for 15 min was stored at −20 °C for subsequent research. The liver, kidney, spleen, heart, lung, and testis were excised to determine the organ coefficient. The organs were washed with sterile PBS and weighed after removing blood and water using an absorbent paper. The organ coefficient was calculated according to Formula (1).

(1) $$OC=\frac{OW}{BW}\times 100\%$$

where OC is the organ coefficient; OW is the organ weight; BW is the body weight.

### 2.4. Analysis of Liver Injury

The activities of alanine aminotransferase (ALT) and aspartate aminotransferase (AST) in serum served as toxicological biomarkers for assessing liver injury [32]. Following the manufacturer’s instructions, serum AST and ALT levels were measured using ALT and AST assay kits (Nanjing Jiancheng Bioengineering Institute, Nanjing, China), and the AST/ALT ratio was calculated [33].

Appropriate amounts of intact liver tissue were taken, washed with 10% formalin, and fixed for more than 24 h, and liver tissue sections were prepared. Liver sections were stained with hematoxylin and eosin (HE) to observe the structure and form of liver histocytes; cellular changes were analyzed using Ridit [34].

### 2.5. Reproductive Injury Analysis

After blood collection, mice were euthanized, and the left epididymis tail was removed immediately. It was immersed in sterile PBS at 37 °C, cut into pieces, and shaken lightly for 1 min to allow the sperm to swim out. It was filtered through a 40-mesh cell strainer to obtain the sperm suspension. The morphology and motility of sperm were observed and analyzed using computer-assisted sperm analysis (ML-810JZ, Nanning Song Jing Tianlun Bio-Technology Co., Ltd., Nanning, China), with the entire operation conducted in an environment maintained at 37 °C and completed within 30 min [35].

Lactate dihydrogen-X (LDH-X) in the testicular tissue was used as a toxicological biomarker of reproductive injury [36]. The testis tissue was homogenized with a sterile glass homogenizer on ice and diluted with sterile PBS. The supernatant was collected following centrifugation at 8000× *g* at 4 °C for 10 min to determine the LDH-X concentration [37]. The LDH-X activity was measured using the LDH isoenzymes assay kit (Nanjing Jiancheng Bioengineering Institute, Nanjing, China) following the manufacturer’s instructions.

### 2.6. Intestinal Tissue Analysis

The intestinal tissue was separated, and the small intestine was cut at the same position at an equal length. After being washed with PBS, the tissues were fixed with 10% formalin for more than 24 h, and the small intestine tissue sections were prepared for HE staining. Tissue sections were measured under a microscope with 40 × combined magnification, using an image processing and analysis system (Version 1, Leica Imaging Systems Ltd., Cambridge, UK). At least 10 well-oriented intact villi and their associated crypts were examined in each intestinal section. Villus height and crypt depth were measured using a light microscope fitted with an image analysis system (AxioScope A1, Carl Zeiss, Jena, Germany), and the mean villus height and crypt depth of each section was calculated [38]. The villus height to crypt depth ratio (V/C) was then calculated according to Formula (2).

(2) $$V/C=\frac{Averagevillusheight}{Averagecryptdepth}\times 100\%$$

where V/C is the ratio of villus height to crypt depth.

### 2.7. Data Analysis

All analyses were conducted in triplicate. Data were processed using SPSS v. 19.0 (IBM Corp., Armonk, NY, USA) and expressed as the mean ± standard deviation. One-way ANOVA followed by a post hoc Dunnett’s test was performed to analyze the difference among groups, and a Ridit analysis (http://spssau.com/indexs.html accessed on 4 December 2023.) was performed to analyze the severity of pathological injury. *p* < 0.05 indicated a statistically significant result.

## 3. Results

### 3.1. Effects of Rumen Microbiota Transplantation on Body Weight and Food Intake of Mice

**Figure 1:**
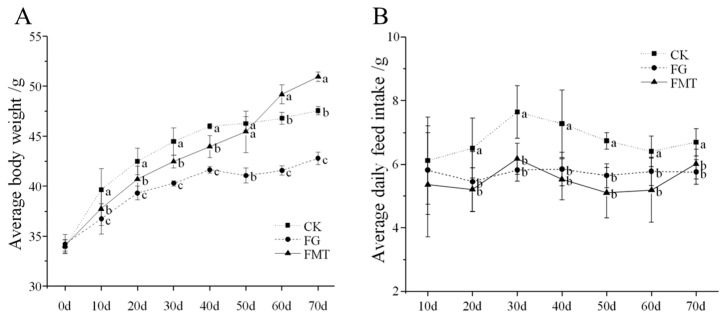
Body weight and food intake of mice. Note: (**A**): Body weight of mice; (**B**): feed intake of mice. Values with different small-letter superscripts indicate a significant difference between groups at the same time (*p* < 0.05).

The effect of rumen microbiota transplantation on the body weight of CM-fed mice is shown in Figure 1A. At the beginning of the experiment (0 d), the body weight of mice in all groups was similar (*p* > 0.05). Throughout the experiment, mice in each treatment group showed varying degrees of weight gain, but the rate of weight gain differed due to differences in feeding conditions. The FG group showed the slowest weight gain (*p* < 0.05), while the CK group showed the fastest weight gain within 50 days (*p* < 0.05). After 50 days, the FMT group showed the fastest weight gain (*p* < 0.05).

The effect of rumen microbiota transplantation on the feed intake of CM-fed mice is shown in Figure 1B. The feed intake of the mice in each group increased initially and then decreased with extended feeding time. There was no difference between the FG group and the FMT group throughout the experiment (*p* > 0.05), but from the 20th day, the feed intake of the FG and FMT groups was lower than that of the CK group (*p* < 0.05).

### 3.2. Effects of Rumen Microbiota Transplantation on the Organ Coefficients of Mice

**Figure 2:**
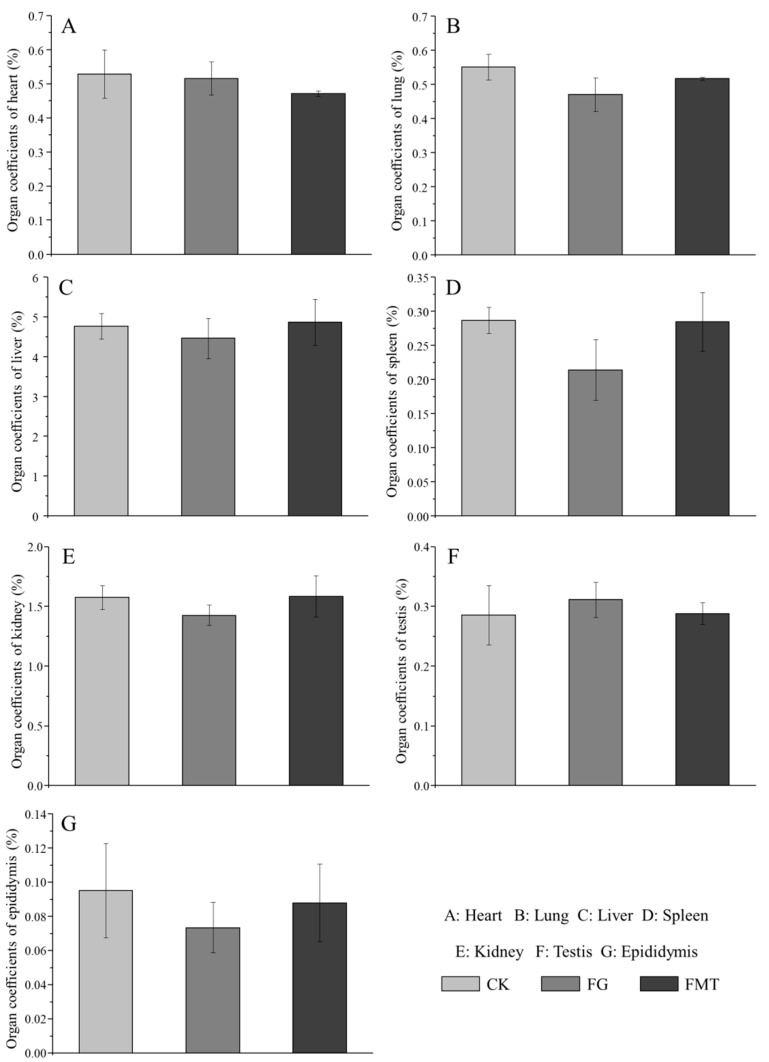
Effects of FMT on organ coefficients in mice. Note: Value columns with the same or no small-letter superscripts mean no significant difference (*p* > 0.05), while those with different small-letter superscripts indicate a significant difference (*p* < 0.05).

The effect of rumen microbiota transplantation on the organ coefficients of CM-fed mice is shown in Figure 2. The organ coefficients of the FG group were lower than those of the CK group. Except for the heart coefficient, the organ coefficients of the FMT group were lower than those of the CK group and higher than those of the FG group. However, there was no difference in the organ coefficients of the mice in each group (*p* > 0.05). Overall, the organ coefficients of the FMT group were closer to those of the CK group compared with the FG group.

### 3.3. Effects of Rumen Microbiota Transplantation on Liver Function in Mice

**Figure 3:**
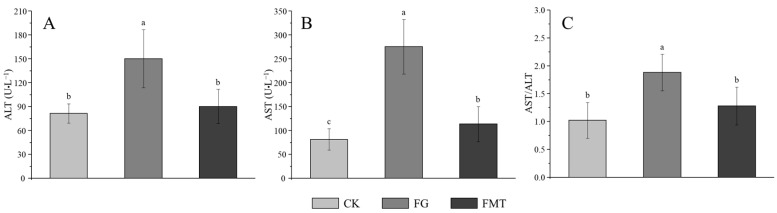
AST and ALT enzyme activities in serum. Note: (**A**): Alanine aminotransferase (ALT), (**B**): aspartate aminotransferase (AST), (**C**): AST/ALT ratio. Value columns with the same or no small-letter superscripts mean no significant difference (*p* > 0.05), while those with different small-letter superscripts indicate a significant difference (*p* < 0.05).

The effect of rumen microbiota transplantation on AST and ALT enzyme activities in the serum of CM-fed mice is shown in Figure 3. The AST and ALT activities and AST/ALT ratio in the FG group were significantly higher than those in the other two groups (*p* < 0.05). The AST in the FMT group was significantly higher compared to the CK group (*p* < 0.05), and there was no difference in the ALT or AST/ALT ratio (*p* > 0.05).

**Figure 4:**
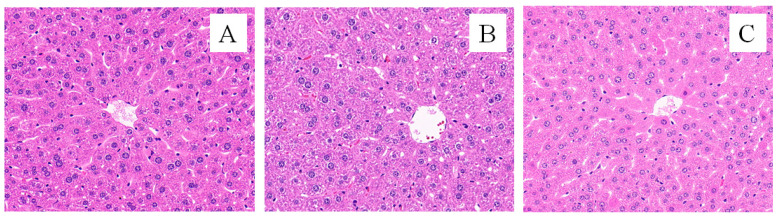
Effects of FMT on liver pathology (HE, ×400). Note: (**A**): CK group; (**B**): FG group; (**C**): FMT group.

The pathological dissection of the liver tissue is shown in Figure 4. The liver morphology in the CK group showed that the liver cells were normal and the hepatic cords and sinusoids were distinct (Figure 4A); in the FG group, cells swelled, the intercellular boundaries were unclear, the cytoplasm was loose and contained pink acidophilic granules and a large number of binuclear cells, and liver cells showed granular degeneration or even hydropic degeneration (Figure 4B). In the FMT group, some liver cells showed enlarged cell volumes; a part of the hepatic cord was blurred, while the hepatic sinusoids were distinct. There were a few binuclear cells, and a few liver cells showed granular degeneration (Figure 4C). There were significant differences in the Ridit values (Table 2) among the three treatment groups (*p* < 0.05), with the FG group showing the most severe liver cell injury, followed by the FMT group, and the CK group exhibiting the healthiest liver cells.

### 3.4. Effect of Rumen Microbiota Transplantation on the Reproductive Function of Mice

**Figure 5:**
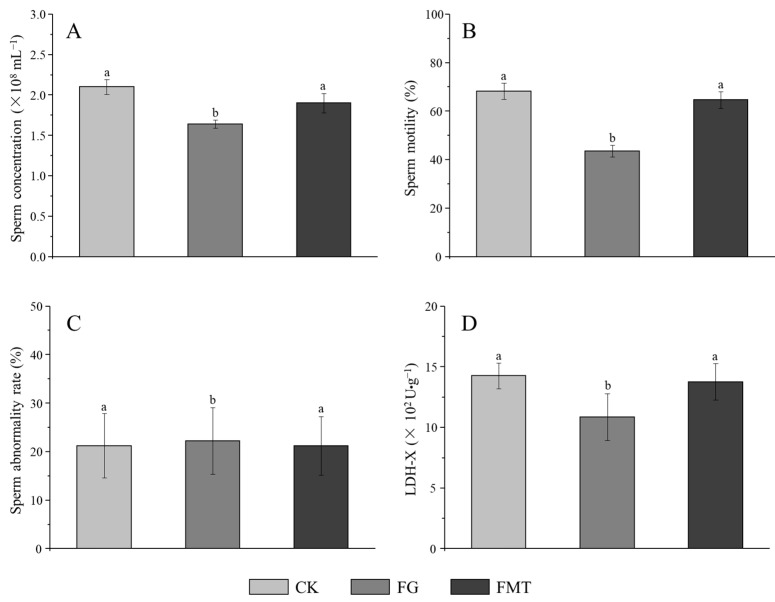
Effects of FMT on the reproductive function of male mice. Note: (**A**): Sperm concentration, (**B**): sperm motility, (**C**): sperm abnormality rate; (**D**): activity of LDH-X in the testis. Value columns with the same or no small-letter superscripts mean no significant difference (*p* > 0.05), while those with different small-letter superscripts indicate a significant difference (*p* < 0.05).

The effect of rumen microbiota transplantation on the reproductive function of CM-fed mice is shown in Figure 5. There was no significant difference in the sperm abnormality rate in each group (*p* > 0.05) (Figure 5C) and sperm concentration and motility between the CK group and FMT group (*p* > 0.05), but both were significantly higher than those in the FG group (*p* < 0.05) (Figure 5A,B). The LDH-X activity in the FMT group was lower than that in the CK group but was statistically insignificant (*p* > 0.05), while the LDH-X activity in the FG group was significantly lower than that in the CK and FMT groups (*p* < 0.05) (Figure 5D).

### 3.5. Effect of Rumen Microbiota Transplantation on the Small Intestine of Mice

**Figure 6:**
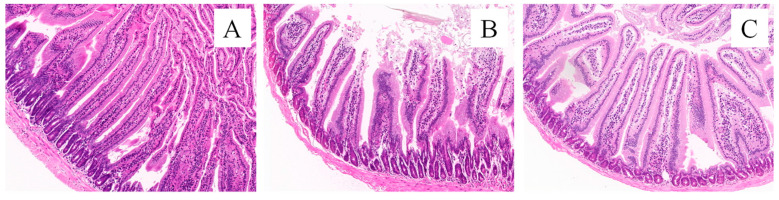
Morphology of the small intestine tissues of mice (HE, ×400). Note: (**A**): CK group; (**B**): FG group; (**C**): FMT group.

**Figure 7:**
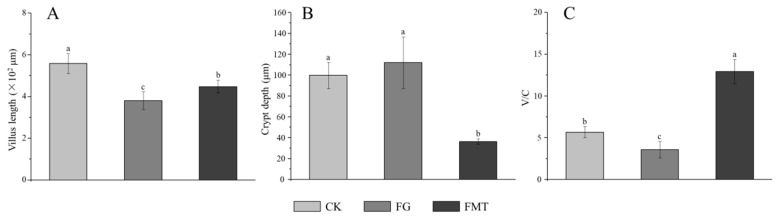
Parameters of small intestine tissue in mice. Note: (**A**): Villus height, (**B**): crypt depth, (**C**): V/C rate. Value columns with the same or no small-letter superscripts mean no significant difference (*p* > 0.05), while those with different small-letter superscripts indicate a significant difference (*p* < 0.05).

Small intestine sections are shown in Figure 6. The villus height and crypt depth are measured according to the pathological sections of the small intestine, and the results are shown in Figure 7. The small intestinal villi in the CK and FMT groups showed a regular and dense arrangement, along with a dense columnar cell arrangement (Figure 6A,C). Small intestinal villi in the FG group were loosely arranged, and the top segment was damaged (Figure 6B). According to the measurement and analysis (Figure 7), there were significant differences in the villus height of each treatment group (*p* < 0.05), and the descending order was CK > FMT > FG. The crypt depth in the FG group showed no difference compared to the CK group (*p* > 0.05), but both were significantly greater than in the FMT group (*p* < 0.05). V/C showed a significant difference among the three groups (*p* < 0.05), and the descending order was FMT > CK > FG.

## 4. Discussion

Changes in body weight and ADFI are important indicators for evaluating the toxicity of compounds to organisms [39]. As an anti-nutritional factor, gossypol reduces the palatability of feeds and decreases animal feed intake [40]. In this study, mice in the FG and FMT groups were fed a gossypol-containing diet, resulting in lower feed intake than the mice in the CK group. Animal feed intake and body weight gain were positively correlated, and the FMT group had lower feed intake than the CK group. The body weight of mice in the FMT group was significantly lower than that in the CK group from 10 to 50 days, consistent with a gossypol diet reducing animal feed intake. Rumen microorganisms have some functions, such as degrading dietary fiber, starch, and protein and synthesizing fatty acids and vitamins [41]. Plateau Pikas obtain beneficial gut microbes from yak intestines by feeding on yak feces [42]. Mice transplanted with Boer goat rumen microbes showed significant weight gain [43]. Beneficial gut microorganisms obtained by FMT mice through the transplantation of rumen microbes likely enhanced their ability to digest and absorb feed. In this study, at the later stage (after 50 days), more beneficial gut microorganisms settled in the intestine over time, resulting in a gradual weight gain in the FMT group, surpassing the weight of mice in the CK group, although their feed intake was lower than that of the CK group.

Changes in organ coefficients are important indicators for judging the health status of tested animals [44]. Gossypol exerts moderate acute toxicity in mice with an oral LD50 of 500–950 mg/kg [45]. In this study, the gossypol content in the diet was 100.06 ± 0.42 mg/kg. Since the gossypol intake during the experiment was within a safe range, there were no significant differences in the organ coefficients among the groups of mice. Rumen microbiota transplanted into the mice in the FMT group posed no threat to their health. However, the organ coefficients of the FG group were lower than those of the CK group, indicating that the long-term intake of low-dose gossypol may adversely affect mice. The long-term intake of low doses of gossypol can accumulate in liver tissue [6] and cause pathological changes in the liver tissue [46]. Probiotics such as *Bacillus amyloliquefaciens*, *Bacillus subtilis*, *Lactobacillus plantarum*, and *Candida dattila* in the rumen of sheep can improve the intestinal micro-ecological environment and immune organ coefficient of mice; protect the liver; and reduce the activity of ALT, AST, and glutamyl transferase (GGT) in serum [41,47]. In this study, the serum ALT and AST levels and AST/ALT in the FMT group were lower than those in the FG group. Through liver tissue section and Ridit analyses, liver damage in the FMT group was found to be lighter than that in the FG group. These indicators suggest that rumen microbiota transplantation can alleviate liver damage induced by gossypol.

Gossypol accumulates in the testis and inhibits the growth of mammalian germ cells, sperm formation, and motility [11,12,48]. Testicular mitochondria are the most sensitive to gossypol. After gossypol exposure, the activity of the mitochondrial marker enzyme LDH-X is inhibited [36]. The gossypol diet led to reproductive damage in the FG group. Short-chain fatty acids (SCFAs) produced by the rumen microbiota can enter the testis through the bloodstream, regulate the testicular microenvironment, and promote spermatogenesis, further improving sperm count and motility [49,50]. Therefore, in the FMT group, after rumen microbiota transplantation, the reproductive damage induced by gossypol was alleviated, and the sperm concentration, sperm motility, and LDH-X activity reached normal levels.

Evaluation indicators such as villus height and crypt depth can reflect intestinal digestive and absorptive function, cell development maturity, and mucosal barrier function [51]. When the villus height increases, the contact area with chyme increases, thereby enhancing the digestive and absorptive functions of the small intestine. In the mature digestive tract, the function of crypt epithelial cells reduces, and the crypt depth becomes shallower. While the V/C ratio increases, the digestive and absorptive functions of the small intestine are increased, and the structure and function of the intestinal mucosa are improved. In this study, compared with the CK group, the FG group showed decreased villus height and enhanced crypt depth, indicating that gossypol feeding is damaging to the small intestinal villi. Rumen microorganisms and their metabolites (SCFAs) can increase the diversity of intestinal microbiota in mice, increase the abundance of beneficial bacteria belonging to the *Lactobacillus* and *Schwartzia* genera, and improve the body’s antioxidant and immune indicators [47,49]. SCFAs can enhance the intestinal immune barrier by controlling the differentiation of regulatory T cells and the bactericidal activity of macrophages [52]. Therefore, after rumen microbiota transplantation, the intestinal immune barrier of the FMT group was improved, the villus height increased, the crypt depth became shallower, and the V/C ratio increased. Rumen microbiota transplantation improved the digestive and absorptive functions of the mouse intestine.

Rumen microbiota can digest and utilize plant materials that monogastric animals cannot [53]. Through microbiota transplantation, unique rumen microbes can be introduced into animals exposed to plant toxins [23]. In this study, male mice fed a gossypol diet showed significantly enhanced tolerance to this harmful plant compound after receiving rumen microbiota transplantation, confirming that rumen microbiota from ruminant animals can mitigate reproductive and liver damage induced by gossypol in monogastric animals. However, rumen microbiota is composed of hundreds of different microorganisms, including bacteria, archaea, fungi, and protozoa, which engage in complex symbiotic relationships [53]. Further research is needed to identify which microorganisms play key roles in detoxifying gossypol. Additionally, rumen microbiota transplantation can influence animal health by altering the recipient’s gut microbiota [54]. Understanding changes in the gut microbiota of treated mice could provide more supportive evidence for this study. Therefore, identifying key rumen microbes and their effects on recipient animal gut microbiota will be crucial for future research on gossypol tolerance in monogastric animals.

## 5. Conclusions

The rumen microbiota from Hu sheep is harmless to recipient mice and can enhance their tolerance to gossypol. Rumen microbiota transplantation alleviates the inhibitory effects of a gossypol diet on feed intake and reduces serum ALT and AST enzyme activities, as well as the AST/ALT ratio, thereby relieving gossypol-induced liver damage. Additionally, it improves sperm concentration and motility in male animals, increases LDH-X activity, and reduces sperm abnormality rates, thus alleviating gossypol-induced reproductive damage in male mice. Rumen microbiota also aids in the recovery of small intestinal villus height, reduces crypt depth, increases the V/C ratio, and helps maintain the structural integrity and weight gain of the recipient’s intestine. Therefore, rumen microbiota may potentially be used to treat gossypol toxicity in animals or serve as a probiotic additive in cottonseed meal diets.

## Figures and Tables

**Table 1:** Grouping and treatment.

Group	Treatment	Description
CK	Fed a gossypol-free diet; oral administration of CMC-Na solution	Blank control group
FG	Fed a gossypol diet (CM diet, FG = 100.06 ± 0.42 mg/kg); oral administration of CMC-Na solution	Negative control group
FMT	Fed a gossypol diet (FG = 100.06 ± 0.42 mg/kg); oral administration of FMT oral solution	Rumen microbiota transplant group

**Table 2:** Pathological changes in the liver and results of the Ridit analysis.

Treatments	n	Injury Level					Score	Ridit-Value
		0	1	2	3	4		
CK	10	8	1	1	0	0	3	7.50 ^c^
FG	10	0	0	3	5	2	29	24.20 ^a^
FMT	10	2	3	4	1	0	14	14.80 ^b^

Note: Values with different small-letter superscripts indicate a significant difference (*p* < 0.05). According to the pathological changes in the liver, the following grading standards were used: Level 0, score 0, normal morphology, no injury observed; Level 1, score 1, number of injured cells does not exceed 1/4 of the total cells; Level 2, score 2, the number of injured cells does not exceed 1/2 of the total cells; Level 3, score 3, the number of injured cells does not exceed 3/4 of the total cells; Level 4, score 4, the number of injured cells exceeds 3/4 of the total cells.

## Data Availability

The data presented in this study are available from the corresponding author upon request. All data generated or analyzed during this study are included in this published article.
